# The effect of cognitive reappraisal and expression suppression on sadness and the recognition of sad scenes: An event-related potential study

**DOI:** 10.3389/fpsyg.2022.935007

**Published:** 2022-09-23

**Authors:** Chunping Yan, Qianqian Ding, Yifei Wang, Meng Wu, Tian Gao, Xintong Liu

**Affiliations:** School of Psychology, Xinxiang Medical University, Xinxiang, China

**Keywords:** sadness, cognitive reappraisal, expressive suppression, recognition, ERP

## Abstract

Previous studies have found differences in the cognitive and neural mechanisms between cognitive reappraisal and expression suppression in the regulation of various negative emotions and the recognition of regulated stimuli. However, whether these differences are valid for sadness remains unclear. As such, we investigated the effect of cognitive reappraisal and expression suppression on sadness regulation and the recognition of sad scenes adopting event-related potentials (ERPs). Twenty-eight healthy undergraduate and graduate students took part in this study. In the regulation phase, the participants were asked to down-regulation, expressive suppression, or maintain their sad emotion evoked by the sad images, and then to perform an immediately unexpected recognition task involving the regulated images. The behavioral results show that down-regulation reappraisal significantly diminished subjective feelings of sadness, but expressive suppression did not; both strategies impaired the participants’ recognition of sad images, and expressive suppression had a greater damaging effect on the recognition of sad images than down-regulation reappraisal. The ERP results indicate that reappraisal (from 300 ms to 1,500 ms after image onset) and expressive suppression (during 300–600 ms) significantly reduced the late positive potential (LPP) induced by sadness. These findings suggest that down-regulation reappraisal and expression suppression can effectively decrease sadness, and that down-regulation reappraisal (relative to expression suppression) is a more effective regulation strategy for sadness. Both strategies impair the recognition of sad scenes, and expression suppression (compared to down-regulation reappraisal) leads to relatively greater impairment in the recognition of sad scenes.

## Introduction

Sadness is a typical negative emotion characterized by sorrow, pain, helplessness, grief, and so on, for instance, the grief felt by those who lost their loved ones due to COVID-19, and the sorrow felt for failing the exam. The level of sadness is a continuum from nothing to grief (An et al., 2017). Sadness affects our bodies and minds and can last from a few seconds to a few hours. Moderate sadness is good for the human body, as it can strengthen one’s coping capacity when dealing with an emergency (Nesse, 1990). However, intense and persistent states of sadness have adverse effects on people’s behavior, and individuals with severe cases may experience depressive symptoms (Zamoscik et al., 2014; Tebeka et al., 2021). Sadness, as a fairly mild negative emotion, has different cognitive and neural mechanisms from those of disgust, anger, and fear (Heining et al., 2000; Kreibig et al., 2007; Urry, 2009). Hence, we examined the regulatory effect of emotion regulation strategies on sadness.

Cognitive reappraisal and expression suppression are the two most commonly used regulation strategies (Dillon et al., 2007; Hayes et al., 2010; Knight and Ponzio, 2013). Cognitive reappraisal is an antecedent-focused emotion regulation strategy occurring in the early stages of the emotional process; it successfully changes emotional experiences through the reinterpretation of emotional events, while expressive suppression is a response-focused emotion regulation strategy that occurs in the late stages of the emotional process by suppressing emotional activities (such as facial expressions) that will happen or are currently transpiring (Gross, 1998a; Gross and Thompson, 2007; Chiao, 2011). Past studies on behavioral performance have explored the effects of cognitive reappraisal and expressive suppression on negative emotions. Cognitive reappraisal can effectively alleviate one’s subjective experiences of negative emotions (such as anger and disgust) (Goldin et al., 2008; Ray et al., 2008; Hofmann et al., 2009) and can also reduce negative expression behavior (Goldin et al., 2008). The effect of expressive suppression on the subjective experiences of negative emotions is controversial. In prior research, suppression resulted in a decrease (Gross and Levenson, 1997; Goldin et al., 2008) or no change (Gross and Thompson, 2007; Vrtićka et al., 2011) in negative subjective ratings. Further, cognitive reappraisal more effectively diminished individuals’ negative subjective feelings in terms of disgust than expression suppression, while expression suppression caused stronger physiological responses (such as, a rise in blood pressure and increased fingertip pulse amplitude) (Gross, 1998a; Goldin et al., 2008; Butler and Gross, 2009). In addition, a few studies explored the effect of expressive suppression on sadness. Gross and Levenson (1997) invited 180 female participants to see sad, neutral, and pleasant film under suppression and non-suppression conditions, and the participants adopting suppression had lower subjective emotional experience. Another research found that the expression suppression of sadness was positively related to depression, and sadness suppression appeared to be a psychosocially adaptive emotional regulation pattern in the Chinese cultural context (Zhou et al., 2016; Dryman and Heimberg, 2018).

Other research has employed event-related potentials (ERPs) to explore brain dynamics associated with emotion generation and regulation. In this sense, the P2 component is early ERP component in emotional processing (Viviani, 2013). P2 reflects the individual’s selective attention to emotional information, and a larger P2 amplitude or a shorter latency indicates the individual’s faster search for, and processing of, visual emotional information (Carretié et al., 2004; LijffIjt et al., 2009; Fisher et al., 2010; Schindler and Bublatzky, 2020). An ERP study found reduced P2 amplitude by reappraisal and suppression, suggesting the two strategies attenuated individuals’ attentional bias of negative stimuli (Wang et al., 2018). P3 (a positive component that occurs approximately 300 ms after stimulus onset) demonstrates the individual’s attention allocation to, and evaluation of, the emotional stimulus, including P3a and P3b (Polich, 1987; Hada et al., 2000). P3a, which has a relatively short peak latency and is primarily distributed in the fronto-central region, denotes attention processing of the stimulus, while P3b (distributed in the central-parietal and parietal areas) is thought to reflect subsequent memory storage (Polich, 2007; Kirihara et al., 2009). LPP (a late positive component) is most commonly employed in emotion regulation studies, and the parietal LPP represents the emotional significance of the stimuli. The more positive LPP showed an enhancement in emotional intensity (Cuthbert et al., 2000; Moser et al., 2009; Yan et al., 2018), which can be utilized as an index of regulation success (Hajcak et al., 2009). In some related studies, down-regulation reappraisal evoked a smaller parietal LPP than the viewing condition (view the images and respond naturally and explicitly) (Moser et al., 2009; Paul et al., 2013; Schönfelder et al., 2014; Shafir et al., 2015), or did not significantly reduce central-parietal LPP (Langeslag and Van Strien, 2010; Baur et al., 2015), and even increased it (Wu et al., 2013; Langeslag and Surti, 2017). An ERP study explored the electrophysiological mechanism in the process of cognitive reappraisal of fearful and sad stimuli, and found that cognitive reappraisal (compared to natural viewing) activated the bilateral frontal–central region in the regulation of the sadness stimulus at 1,500–2,500 ms; next, the regulation of the fear stimulus and the sadness stimulus activated the left frontal–central region at 2,500–4,000 ms (Wei et al., 2021). Additionally, expressive suppression significantly decreased LPP of fear during 450–550 ms (Cheng et al., 2011) or of unpleasant emotion during 350–600 ms (Moser et al., 2006).

To date, only a few studies have used ERP to compare the regulation effects of cognitive reappraisal and expression suppression on negative emotions (Cheng et al., 2011; Paul et al., 2013; Wang et al., 2018). Paul et al. (2013) scrutinized the temporal characteristics of cognitive reappraisal, expressive suppression, and distraction using ERPs, and all three strategies successfully decreased the LPP and self-reported negative effects on negative emotions. Expressive suppression and distraction influenced emotional responses earlier than cognitive reappraisal; it was thought that expressive suppression (preventively used) might disrupt the emotion-generative process from the very beginning instead of targeting the emotional response itself. In an ERP study on the regulation of fear, cognitive reappraisal significantly lowered the average LPP amplitude during the time window of 400–450 ms and 550–600 ms compared with the expressive suppression and control groups; cognitive reappraisal and expression suppression significantly reduced the average LPP amplitude during the time window of 450–550 ms compared with the control group; therefore, cognitive reappraisal played an earlier role in the regulation of fear than expression suppression and lasted longer as well (Cheng et al., 2011). Another ERP study (Wang et al., 2018) on negative emotions showed that both cognitive reappraisal and expression suppression effectively down-regulated negative subjective ratings, but cognitive reappraisal down-regulated negative ratings more effectively than suppression. According to the ERP results, cognitive reappraisal and expressive suppression significantly reduced P2 and the LPP amplitude than the negative viewing condition. However, expressive suppression down-regulated LPP more effectively than cognitive reappraisal. The conflict between self-reported outcomes and ERP results suggests that self-reported outcomes may have subjective bias, so objective research methods should be used in studies on emotion regulation. Hence, there are different temporal characteristics in the cognitive and neural mechanisms between cognitive reappraisal and expression suppression in the regulation of different kinds of negative emotions. Notwithstanding, whether these differences are valid for sadness remains unclear. As such, for the present study, we investigated the effects and neural mechanisms of cognitive reappraisal and expression suppression on sadness using ERPs.

In addition, emotion regulation strategies such as cognitive reappraisal and expression suppression had effects on memories of regulated emotional stimuli. Cognitive reappraisal (up- or downregulation) enhances memories of emotional stimuli compared with passive viewing (view the images and respond naturally and explicitly) (Richards et al., 2003; Dillon et al., 2007; Hayes et al., 2010; Kim and Hamann, 2012; Yeh et al., 2019). Dillon et al. (2007) found that compared to passive viewing, both the up-regulation and down-regulation conditions improved the recall of negative pictures. However, down-regulation reappraisal may have impaired recognition performance for negative stimuli (Knight and Ponzio, 2013; Leventon and Bauer, 2015). Knight and Ponzio (2013) required participants to use cognitive reappraisal to increase and decrease negative and positive emotions evoked by pictures. They found that up-regulation reappraisal strengthened recall accuracy, but down-regulation reappraisal reduced recall accuracy for emotional pictures. Emotion regulation involved the two processes of emotion generation and emotion regulation, which would have some effect on later memories (Deng et al., 2021). We thought that cognitive reappraisal would have the potential to modulate memory through elaboration, attentional deployment, and arousal, as up-regulation reappraisal facilitates memory performance that is potentially due to heightened elaboration, arousal, and attention to emotional stimuli. However, down-regulation of negative emotions led to reduced visual attention to negative scenes and lower negative arousal, which evoked a decrease in memory performance. Nevertheless, deep stimulus elaboration contributed to memory enhancement; therefore, the effect of down-regulation reappraisal on the memories of emotional stimuli presents an inconsistency (Knight and Ponzio, 2013; Leventon and Bauer, 2015). Moreover, expressive suppression could impair memories of emotional events relative to the passive viewing condition (Richards and Gross, 2000; Bonanno et al., 2004; Dillon et al., 2007; Hayes et al., 2010; Binder et al., 2012), which may be related to the masking and monitoring of emotional displays, the process probably diverting finite attentional resources away from elaborating on emotional events (Richards and Gross, 2000; Richards et al., 2003; Dillon et al., 2007). Alternatively, suppression might guide an individual to selectively focus on less emotionally arousing elements of stimuli (Bebko et al., 2011; Knight and Ponzio, 2013). At present, it remains unclear whether the mechanisms of cognitive reappraisal and expressive suppression on the memories of sad scenes produce different memory outcomes. Thus, we also investigated the effects of cognitive reappraisal and expression suppression on the recognition of sad scenes in this study.

Most emotion regulation studies have focused on the down-regulation of negative emotions because it has clear clinical relevance (Hajcak and Nieuwenhuis, 2006; Blechert et al., 2012; Zhang et al., 2012; Moser et al., 2014), for instance, alleviating anxiety, thus we adopted down-regulation as the cognitive reappraisal method for sadness. We manipulated two emotion regulation strategies—down-regulation reappraisal and expressive suppression—and view condition as the baseline. We mainly examined the effects and neural mechanisms of down-regulation reappraisal and expression suppression on sadness using ERPs, and we also explored the effect of the two strategies on the recognition of regulated sad scenes. This study consisted of emotion regulation and test phases; in the emotion regulation phase, we employed a trial-by-trial manipulation of instructions to avoid potential confounds present in block designs (Moser et al., 2009). Sadness is a fairly mild negative emotion different from fear and so on (Heining et al., 2000; Kreibig et al., 2007; Urry, 2009), and sadness suppression is a psychosocially adaptive emotional regulation pattern in the Chinese cultural context (Zhou et al., 2016; Dryman and Heimberg, 2018), so we predicted that, down-regulation reappraisal and expressive suppression would significantly reduce subjective feelings of sadness, and down-regulation and expressive suppression would significantly lower the LPP amplitude induced by sadness (Cheng et al., 2011; Wang et al., 2018), and down-regulation reappraisal would work more than expressive suppression in the early stages (Cheng et al., 2011); down-regulation reappraisal would enhance recognition performance due to the previous studies (Richards et al., 2003; Dillon et al., 2007; Hayes et al., 2010; Kim and Hamann, 2012; Yeh et al., 2019), probably through heightened attention and elaboration on the sad images (Knight and Ponzio, 2013), but expressive suppression would reduce recognition performance (Richards and Gross, 2000; Bonanno et al., 2004; Dillon et al., 2007; Hayes et al., 2010; Binder et al., 2012), therefore, we also predicted that down-regulation reappraisal would enhance the P2, P3a, and P3b than view-sad condition, but expressive suppression would decrease the P2, P3a, and P3b than view-sad condition.

## Materials and methods

### Participants

Twenty-eight healthy undergraduate and graduate students from Xinxiang Medical University participated in this study (13 were male; all were between 18 and 25 years old, mean age = 21.32 ± 2.11). The sample size was determined according to the results of a power analysis run through G-Power software (power > 0.8, α = 0.05) (Faul et al., 2007), which suggested that a minimum sample size of *n* ≥ 28 was required for that purpose. All participants were right-handed and had normal or corrected-to-normal vision. This study was approved by the institutional ethics committee of Xinxiang Medical University in China, and all methods were carried out according to relevant guidelines. No vulnerable populations were involved. Each participant signed an informed consent form before the formal experiment in accordance with the Declaration of Helsinki.

### Materials

The target stimuli consisted of 240 color images (180 sad and 60 neutral) selected from the Chinese Affective Picture System (CAPS) (Lu et al., 2005), the International Affective Picture System (IAPS) (Lang et al., 2010), and the internet. Sad pictures, for example, a couple who has experienced the earthquake are crying bitterly in the rubble, holding their dead child; neutral pictures, for example, two workers are working hard in the factory. All pictures were uniform in size and resolution ratio through a few treatments adopting Adobe Photoshop 13.1.3. Twenty college students who did not attend the formal experiment provided valence (1 = very not happy, 9 = very cheerful) and arousal (1 = very calm, 9 = very excited) ratings of these images; they also provided sadness ratings (1 = very not sad, 5 = very sad) of the sad images.

The emotional valence of the sad and neutral images was 3.06 ± 0.04 and 4.99 ± 0.04, respectively, and the arousal of the sad and neutral images was 5.61 ± 0.05 and 4.54 ± 0.06, respectively. There were significant differences between the two types of images [valence: *t*_(238)_ = −31.87, *P* < 0.001; arousal: *t*_(238)_ = 14.38, *P* < 0.001]. Among them, 160 images (120 sad and 40 neutral) were used as study (old) items, and 80 images (60 sad and 20 neutral) were used as test (new) items. In addition, all sad images were divided into three groups, respectively, used in the down-regulation reappraisal, expressive suppression, and view conditions, each group including 40 images, and these six groups [3 (down-regulation, view-sad, and expressive suppression) × 2 (old and new)] of sad images had equal valence, arousal, and sadness ratings by statistical tests; the neutral images were used in the view condition, and old and new groups of neutral images also had equal valence and arousal ratings (see the Supplementary File). The subjective ratings of the images in each group are given in Table 1.

**TABLE 1:** The average valence, sadness, and arousal ratings of the stimuli groups in this study.

	Stimuli	View-sad	Down-regulation reappraisal	Expressive suppression	View-neutral
Valence	Regulated	3.06 ± 0.08	3.06 ± 0.08	3.07 ± 0.08	4.99 ± 0.08
	New	3.05 ± 0.12	3.06 ± 0.12	3.04 ± 0.12	5.00 ± 0.12
Arousal	Regulated	5.60 ± 0.09	5.60 ± 0.09	5.60 ± 0.09	4.54 ± 0.09
	New	5.62 ± 0.13	5.62 ± 0.13	5.62 ± 0.13	4.54 ± 0.13
Sadness	Regulated	2.96 ± 0.08	2.95 ± 0.08	2.95 ± 0.08	
	New	2.94 ± 0.11	2.94 ± 0.11	2.94 ± 0.11	

The data after “±” are the standard errors of the mean.

Participants performed the regulation and test task in a quiet, sound-proof room with their eyes 70 cm away from a computer monitor, and each image (1,024 × 768 pixels) was presented in the center of the screen adopting Presentation 0.71 (Neurobehavioral Systems, Albany, CA, United States), with a viewing angle of 16.44 × 15.93^°^.

### Procedure

The participants were told that they would take part in an emotion regulation experiment in which they were required to regulate their emotions as requested. They were trained to follow one of the three instructions during each trial in the down-regulation reappraisal, expressive suppression, and view conditions, respectively. For the down-regulation reappraisal trials, the participants were instructed to imagine the depicted situation getting better (such as the individual in the images soon getting relief) (Chiao, 2011) or to view the images from a detached, third-person perspective and to imagine that the events in the images were fake or made up (Gross, 1998a). For the expressive suppression trials, they were instructed to carefully watch the images while feeling the emotion of the protagonist in the images; not to reveal their feelings (i.e., to suppress their true feelings when watching the images, not letting others see that they had any emotional response); and to try to keep a neutral face throughout the watching process (Richards and Gross, 2000). For the view trials, they were instructed to view the images and respond naturally and explicitly, and to not alter their natural response (Paul et al., 2013). Before the formal experiment, the participants were made familiar with the experimental procedure and keystroke responses through practice.

**FIGURE 1:**
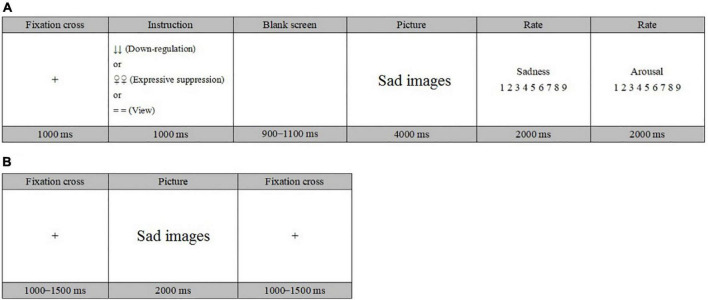
Schematic representations of a trial in the regulation phase **(A)** and the test phase **(B)**.

The formal experiment included four blocks, each containing 40 trials, and three regulation strategies appeared alternately. The experimental flow is shown in Figure 1. Each trial began with a cross fixation point presented for 1,000 ms. Following the fixation, a down-regulation reappraisal cue (↓↓), an expressive suppression cue (♀♀), or a view cue (= =) was displayed on the screen for 1,000 ms; then, the cue was replaced by a blank screen for 900–1,100 ms. After that, an image was presented for 4,000 ms, and the participants were asked to regulate their emotions according to the previous regulatory cues. Next, the participants were asked to evaluate the sadness and arousal ratings they felt about the images within 2,000 ms after the presentation of the image. In the sadness ratings, 1 represented “not sad at all,” 3 signaled “a little sad,” 5 denoted “sad,” 7 indicated “very sad,” and 9 suggested “extremely sad.” In the arousal ratings, 1 represented “very calm,” 3 denoted “a little excited,” 5 referred to “excited,” 7 indicated “very excited,” and 9 meant “extremely excited.” At the end of each block, the participants were asked to give a 6-point subjective rating from 1 (no effort) to 6 (very hard) about their own efforts regarding emotion regulation in this block. There was a 2-min break between the two regulation blocks.

After emotion regulation, the participants were asked to perform an unexpected recognition task involving the regulated images. In each test trial, a cross fixation was first presented for 1,000–1,500 ms, followed by an image for 2,000 ms. The participants were asked to perform an old/new judgment and indicate their response by pressing “F” or “J” on the keyboard within the 2,000 ms, and they were asked to make quick and accurate judgments. The formal test phase included four blocks, and there was a 2-min break between the two test blocks. The average time of the participants in performing the regulation and test task was about an hour.

### Event-related potential recordings and analysis

Electroencephalographic (EEG) data were recorded using a 64-channel Neuroscan amplifier (Brain Products GmbH; Munich, Germany) at a 500 Hz sampling rate with a 0.05–100 Hz bandpass filter. The electrode locations conformed to the extended international 10–20 system. The electrooculogram (EOG) was recorded with two pairs of electrodes: one pair placed above and below the left eye and another pair at the outer canthi of both eyes. All electrodes were referenced online to the left mastoid and re-referenced offline to the average of the right and left mastoid recordings. EOG blink artifacts were corrected using a linear regression estimate (Picton et al., 2000; Hornberger et al., 2004). EEG/EOG signals (impedance < 5 kΩ) were digital bandpass filtered at 0.05–40 Hz and corrected to a 200 ms pre-stimulus baseline. The time range of EEG analysis was 4,000 ms, and trials with a voltage exceeding ± 100 μV were excluded from the ERP analysis.

P2 was defined as the average amplitude during 150–250 ms in frontal area (F3, Fz, and F4) (Hess et al., 1995; LijffIjt et al., 2009), and P3 was defined as the average amplitude during 350–500 ms in frontal area (F3, Fz, and F4) for P3a and central-parietal electrodes (CP3, CPz, and CP4) for P3b (Polich, 1987; Hada et al., 2000), and LPP was defined as the average amplitude in 300–600 ms, 600–1,000 ms, and 1,000–1,500 ms windows in parietal area (P1, Pz, and P2) mainly (Paul et al., 2013; Wei et al., 2021).

Additionally, in statistical analyses of behavioral data, the sadness and arousal ratings of the sad images were compared between three regulation strategies (down-regulation reappraisal, expressive suppression, and view-sad) in the regulation phase, and the recognition discrimination Prs (the hit rate of the old image minus the false alarm rate of the new image) (Snodgrass and Corwin, 1988) and response time were compared between the three regulation strategies in the test phase.

Repeated measures ANOVAs were corrected using the Greenhouse–Geisser method when sphericity is significant. The significance level was 0.05. Multiple comparisons and simple effect analyses were corrected using the Bonferroni correction. All data analyses were conducted with SPSS 18.0 software.

## Results

### Behavioral data

Participants’ regulation ratings and recognition performances are given in Table 2.

**TABLE 2:** The average sadness, arousal ratings, recognition accuracy Prs, and recognition RTs (ms) of the stimuli groups in this study.

	Down-regulation reappraisal	Expressive suppression	View-sad
Sadness	3.71 ± 1.50	4.57 ± 1.50	4.70 ± 1.60
Arousal	3.68 ± 1.40	4.41 ± 1.64	4.45 ± 1.77
Prs	0.64 ± 0.10	0.61 ± 0.13	0.68 ± 0.13
RTs	1,031 ± 162	1,050 ± 143	1,005 ± 155

The data after “±” are the standard deviations of the mean.

### Behavioral data in the regulation phase

**FIGURE 2:**
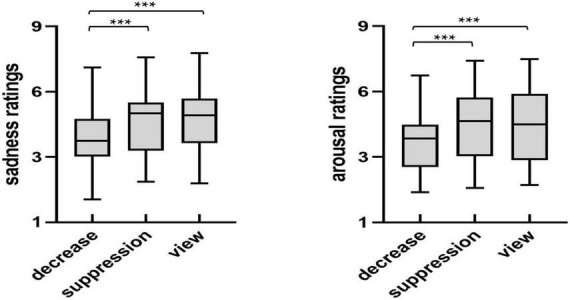
The sadness and arousal ratings of sad images after regulation. *p < 0.05, **p < 0.01, ***p < 0.001.

In order to investigate the differences in the sadness and arousal ratings of the sad images between the regulation strategies, we used a repeated measures ANOVA on the sadness and arousal ratings of the sad images (see Figure 2) with the regulation strategy (down-regulation reappraisal, expressive suppression, and view-sad) as within-subject factors.

The analysis of sadness and arousal ratings showed significant main effects of regulation strategy [sadness: *F*_(2,54)_ = 17.28, *p* < 0.001, η*_*p*_*^2^ = 0.39; arousal: *F*_(2,54)_ = 11.93, *p* < 0.001, η*_*p*_*^2^ = 0.31]. Furthermore, multiple comparisons found that the sadness and arousal ratings of participants under the down-regulation condition were significantly lower than those under the expressive suppression and view-sad conditions (*p* < 0.001, *p* < 0.001, *p* = 0.002, *p* < 0.001). However, there were no significant differences between the expression suppression and view-sad conditions (*p* = 1.000, *p* = 1.000). In summary, down-regulation significantly reduced subjective feelings of sadness and arousal, but expressive suppression did not.

### Behavioral data in the recognition phase

**FIGURE 3:**
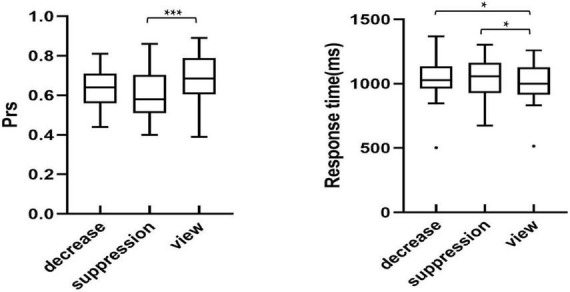
Recognition performance across conditions in the test phase. *p < 0.05, **p < 0.01, ***p < 0.001.

In order to investigate the differences in the recognition of the sad images between the regulation strategies, univariate repeated measures ANOVA was performed with recognition discrimination Prs (the hit rate of the old image minus the false alarm rate of the new image) and response time as the dependent variables, and the regulation strategy (down-regulation reappraisal, expressive suppression, and view-sad) as the independent variable. The participants’ recognition performance is described in Figure 3.

Analysis of Pr showed a significant main effect of regulation strategy [*F*_(2,54)_ = 9.92, *p* < 0.001, η*_*p*_*^2^ = 0.27]. Further multiple comparisons suggested that the participants’ Prs, only under expressive suppression, were significantly lower than that of the view-sad condition, indicating that expressive suppression impaired participants’ recognition of sad images (*p* < 0.001), but there were no significant differences in the Prs between down-regulation reappraisal and the view-sad conditions (*p* = 0.104) or between the down-regulation and expressive suppression conditions (*p* = 0.132).

Analysis of response time revealed that a significant main effect of regulation strategy [*F*_(2,54)_ = 5.93, *p* = 0.008, η*_*p*_*^2^ = 0.18]. Further multiple comparisons found that the participants’ response times under the down-regulation and expressive suppression conditions were significantly slower than those under the view-sad condition (*p* = 0.034, *p* = 0.010), but there was no significant difference between the down-regulation and expressive suppression conditions (*p* = 0.663), indicating that the participants’ memories of sad images were impaired by the regulation of down-regulation reappraisal and expressive suppression. In conclusion, the use of down-regulation reappraisal and expressive suppression significantly increased the recognition time of sad images during the emotion regulation stage, suggesting that down-regulation reappraisal and expressive suppression impaired the participants’ memories of sad images.

### Event-related potential data

**FIGURE 4:**
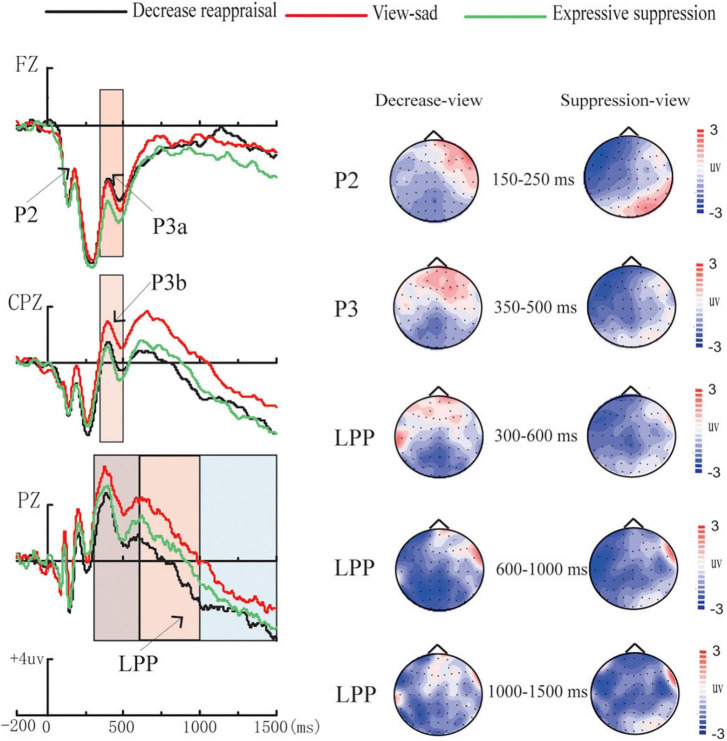
Amplitude distribution and topographic map of ERPs during regulation. Amplitude distribution and topographic map of ERP measurements involving the effects of down-regulation reappraisal and expressive suppression on the regulation of sadness.

**FIGURE 5:**
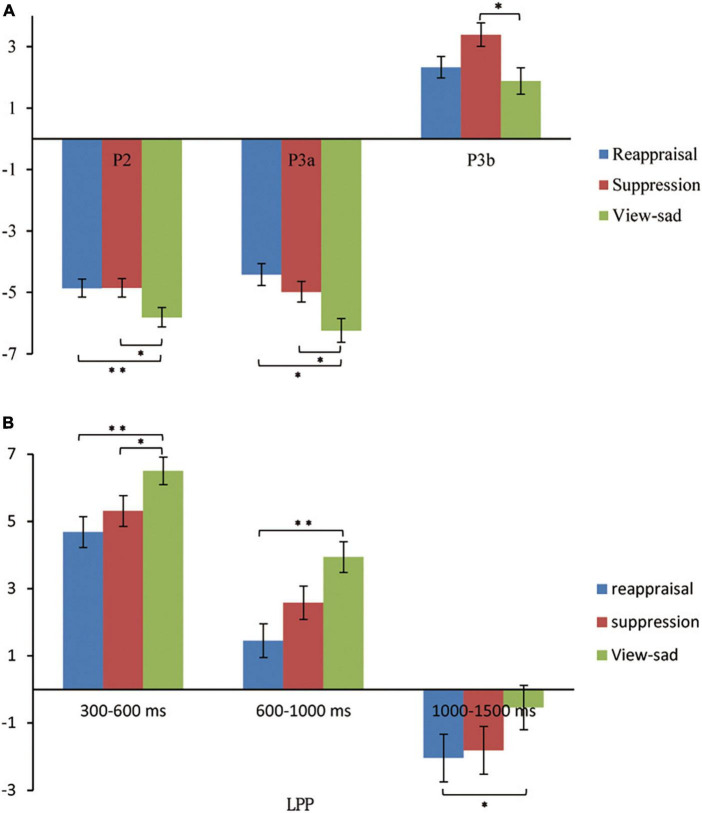
The bar graphs about the differences between the conditions for each ERPS component. **(A)** The graph shows the differences between conditions for P2, P3a, and P3b components. **(B)** The graph shows the differences between conditions for LPP component during 300–600 ms, 600–1,000 ms, and 1,000–1,500 ms. *p < 0.05, ^**^p < 0.01, ^***^p < 0.001.

In order to explore the differences in the average amplitudes between the regulation strategies, the average amplitudes for each condition during each time window were analyzed using repeated measures ANOVAs. Table 3 presents the ERP average amplitudes under each regulation conditions. Figure 4 illustrates the ERP average amplitude distributions and topographic map in the regulation phase. Figure 5 illustrates the differences between the conditions for each ERPS component using bar graphs.

**TABLE 3:** The ERP average amplitude under each regulation condition.

	Time windows	Down-regulation reappraisal	Expressive suppression	View-sad
P2	150–250 ms	−4.86 ± 3.12	−4.85 ± 3.20	−5.81 ± 3.15
P3a	350–500 ms	−4.42 ± 3.79	−4.98 ± 3.56	−6.24 ± 4.12
P3b	350–500 ms	2.33 ± 3.69	3.39 ± 4.03	1.88 ± 4.56
LPP	300–600 ms	4.68 ± 3.80	6.50 ± 4.28	5.31 ± 4.84
	600–1,000 ms	1.45 ± 4.47	3.94 ± 4.85	2.58 ± 5.27
	1,000–1,500 ms	−2.04 ± 6.96	−0.54 ± 6.95	−1.81 ± 7.47

The data after “±” are the standard deviations of the mean.

**P2** (150–250 ms). The average amplitudes were analyzed using 3 (regulation strategy: down-regulation reappraisal, expressive suppression, and view-sad) × 3 (electrode location: left, middle, and right) repeated-measure ANOVAs with the two factors as within factors. The ANOVA revealed non-significant main effects of regulation strategy and electrode location [*F*_(2,54)_ = 2.36, *p* = 0.110, η*_*p*_*^2^ = 0.08, *F*_(2,54)_ = 0.55, *p* = 0.528, η*_*p*_*^2^ = 0.020], and a significant regulation strategy × electrode location interaction [*F*_(4,108)_ = 3.70, *p* = 0.007, η*_*p*_*^2^ = 0.12]. Further simple effect analysis found that the average amplitudes under the down-regulation reappraisal and view-sad conditions were more positive than those under expressive suppression only at electrode F3 (*p* = 0.009, *p* = 0.015).

**P3a** (350–500 ms). The average amplitudes in frontal area were analyzed using 3 (regulation strategy: down-regulation reappraisal, expressive suppression, and view-sad) × 3 (electrode location: left, middle, and right) repeated measures ANOVAs with the two factors as within factors. The ANOVA revealed only one significant main effect of regulation strategy [*F*_(2,54)_ = 5.54, *p* = 0.006, η*_*p*_*^2^ = 0.17], but no significant main effect of electrode location [*F*_(2,54)_ = 0.79, *p* = 0.419, η*_*p*_*^2^ = 0.03] and no significant regulation strategy × electrode location interaction [*F*_(4,108)_ = 0.15, *p* = 0.147, η*_*p*_*^2^ = 0.07]. Further multiple comparisons found that the average amplitudes under the down-regulation reappraisal and view-sad conditions were significantly more positive than those under expressive suppression (*p* = 0.020, *p* = 0.043), and there was no significant difference between the down-regulation reappraisal and view-sad conditions (*p* = 1.000).

**P3b** (350–500 ms). The average amplitudes in central-parietal area were analyzed using 3 (regulation strategy: down-regulation reappraisal, expressive suppression, and view-sad) × 3 (electrode location: left, middle, and right) repeated measures ANOVAs with the two factors as within factors. The results revealed two significant main effects of regulation strategy and electrode location [*F*_(2,54)_ = 5.23, *p* = 0.008, η*_*p*_*^2^ = 0.16; *F*_(2,54)_ = 9.91, *p* < 0.001, η*_*p*_*^2^ = 0.27], but no significant regulation strategy × electrode location interaction [*F*_(4,108)_ = 1.82, *p* = 0.161, η*_*p*_*^2^ = 0.06]. Further multiple comparisons found that the average amplitudes under the view-sad condition were significantly more positive than those under the expressive suppression condition (*p* = 0.016), and the average amplitudes at CP3 and CP4 were significantly more positive than those at CPz (*p* = 0.001, *p* = 0.001).

### Late positive potential

The average amplitudes during 300–600 ms, 600–1,000 ms, and 1,000–1,500 ms were analyzed, respectively, using 3 (regulation strategy: down-regulation reappraisal, expressive suppression, and view-sad) × 3 (electrode location: left, middle, and right) repeated measures ANOVAs with the two factors as within factors.

300–600 ms. The ANOVA revealed the significant main effects of regulation strategy and electrode location [*F*_(2,54)_ = 6.24, *p* = 0.004, η*_*p*_*^2^ = 0.19, *F*_(2,54)_ = 25.67, *p* < 0.001, η*_*p*_*^2^ = 0.49], and a non-significant regulation strategy × electrode location interaction [*F*_(4,108)_ = 2.28, *p* = 0.065, η*_*p*_*^2^ = 0.08]. Further multiple comparisons suggested that the average amplitudes under the down-regulation reappraisal and expressive suppression were more negative than those under view-sad condition (*p* = 0.005, *p* = 0.050), and the average amplitudes at P1 and P2 were significantly more positive than those at Pz (*p* < 0.001, *p* < 0.001).

600–1,000 ms. The ANOVA indicated the significant main effects of regulation strategy and electrode location [*F*_(2,54)_ = 8.05, *p* = 0.001, η*_*p*_*^2^ = 0.23, *F*_(2,54)_ = 25.19, *p* < 0.001, η*_*p*_*^2^ = 0.48], and a non-significant regulation strategy × electrode location interaction [*F*_(4,108)_ = 1.97, *p* = 0.105, η*_*p*_*^2^ = 0.07]. Further multiple comparisons suggested that the average amplitudes under the down-regulation reappraisal were more negative than those under view-sad condition (*p* = 0.001), and the average amplitudes at P1 and P2 were significantly more positive than those at Pz (*p* < 0.001, *p* < 0.001).

1000–1,500 ms. The ANOVA revealed a marginal significant main effect of regulation strategy [*F*_(2,54)_ = 3.08, *p* = 0.054, η*_*p*_*^2^ = 0.10] and a significant main effect of electrode location [*F*_(2,54)_ = 23.91, *p* < 0.001, η*_*p*_*^2^ = 0.47], and a non-significant regulation strategy × electrode location interaction [*F*_(4,108)_ = 1.32, *p* = 0.270, η*_*p*_*^2^ = 0.05]. Further multiple comparisons revealed that the average amplitudes under the down-regulation reappraisal were more negative than those under view-sad condition (*p* = 0.045), and the average amplitudes at P1 and P2 were significantly more positive than those at Pz (*p* < 0.001, *p* < 0.001).

## Discussion

This study explored the effects of cognitive reappraisal and expressive suppression on the regulation of sadness and the recognition of sad scenes. Participants used the down-regulation reappraisal, expressive suppression, and view-sad conditions to regulate sadness in the regulation phase and then performed an unexpected recognition test. Behavioral results showed that down-regulation significantly reduced subjective feelings (including sadness and arousal ratings) of sadness, but expressive suppression did not. Both down-regulation reappraisal and expressive suppression impaired the participants’ recognition of sad images, and expressive suppression had a more harmful effect on the recognition of sad images than down-regulation reappraisal. The ERP data demonstrated that both down-regulation reappraisal and expressive suppression significantly reduced the LPP amplitude induced by sadness, but down-regulation reappraisal works lasting longer than expressive suppression in sadness regulation.

Behavioral results indicated that the participants’ arousal and sadness ratings under the down-regulation reappraisal condition were significantly lower than those under the view-sad and expressive suppression conditions, suggesting that down-regulation reappraisal significantly reduced the sad and arousal level of sadness, but not in line with our expectations, expressive suppression did not alleviate subjective feelings of sadness. The reduction in the subjective ratings of sadness *via* down-regulation reappraisal is consistent with many previous studies on negative emotions (involving disgust, anxiety, and anger) (Goldin et al., 2008; Ray et al., 2008; Hofmann et al., 2009). The effect of expressive suppression on subjective experiences of negative emotions is controversial, with some results highlighting a decrease (Gross and Levenson, 1997; Goldin et al., 2008) and some results finding no decrease (Gross and Thompson, 2007; Vrtićka et al., 2011) in subjective feelings *via* expressive suppression. Consistent with the latter outcomes, our study showed that expressive suppression did not reduce subjective feelings of sadness. The above behavioral results of down-regulation reappraisal, but not expressive suppression, were supported by our ERP findings. We focused more on ERPs than on self-reported emotional feelings because self-reports of emotional experiences might not be sensitive enough (Sheppes and Meiran, 2007; Urry, 2009).

The ERP outcomes revealed that the down-regulation reappraisal and view-sad conditions elicited a larger P2 component than expressive suppression at electrodes F3 during 150–250 ms as we had expected, indicating that the down-regulation reappraisal and view-sad conditions may evoke the individual’s earlier selective attention and processing to image information than expressive suppression (LijffIjt et al., 2009). It could also be that expressive suppression inhibited the input of attentional resources from an early stage. Wang et al. (2018) found that reduced P2 amplitude by reappraisal and suppression may suggest the two strategies attenuated individuals’ attentional bias of negative stimuli; however, in this study, we found only the reduced P2 amplitude by expressive suppression, and no significant difference of P2 component between the down-regulation reappraisal and view-sad conditions, which was not in line with our expectations, indicating that reappraisal and view-sad conditions may occupy certain cognitive resources to recognize situation’s meaning of sad pictures, but expressive suppression may cost less attention resources in the early processing of sad images. Moreover, sad stimuli can activate the left lingual gyrus, left amygdala, and left prefrontal cortex (Batty and Taylor, 2003; Levesque et al., 2003), and the left fronto-central region has an important role in the downregulation of sadness (Wei et al., 2021). Given prior ERP studies, P3a distributed in the fronto-central area represents attention processing to the stimulus, and P3b distributed in the parietal area reflects subsequent memory storage (Polich, 2007; Kirihara et al., 2009). Not in line with our expectations, we only found a significantly increased P3a in the frontal area during 350–500 ms under the down-regulation reappraisal and view-sad conditions than under expressive suppression, combined with the increased left-frontal P2 during 150–250 ms under the down-regulation and view-sad conditions than expressive suppression. The two increased components imply that the down-regulation reappraisal and view-sad conditions required more cognitive resources to regulate or maintain sadness than expressive suppression during 150–250 ms and 350–500 ms. In addition, we found that frontal P3a and centro-parietal P3b decreased under the expressive suppression condition compared to the view-sad condition, indicating that expressive suppression inhibited the input of attentional resources and evoked a memory storage reduction.

The ERP results also showed that down-regulation reappraisal (lasting from 300 ms to 1,500 ms after images onset) and expressive suppression (during 300–600 ms) significantly reduced the LPP amplitude in the parietal region, which were in line with our expectations. The parietal LPP mainly revealed the intensity of emotional stimuli (Cuthbert et al., 2000; Moser et al., 2009; Cauwenberge et al., 2017; Yan et al., 2018). As such, the LPP reduction under the down-regulation reappraisal (from 300 ms to 1,500 ms) and expressive suppression conditions (only during 300–600 ms) signals that down-regulation reappraisal and expressive suppression can effectively mitigate the intensity of sad emotions, and down-regulation reappraisal works lasting longer than expressive suppression. An ERP study about the regulation of fear *via* cognitive reappraisal and expressive suppression showed that the reappraisal group significantly reduced the LPP amplitude in the time windows of 400–450 ms and 550–600 ms compared with the control group, while cognitive reappraisal and expressive suppression significantly reduced the LPP amplitude in the time window of 450–550 ms compared to the control group. This means that cognitive reappraisal has an earlier effect than expressive suppression and lasts longer when it comes to fear (Cheng et al., 2011). Another ERP study showed that cognitive reappraisal (compared to natural viewing) reduced the LPP during 1,500–2,500 ms induced by the sadness stimulus, and also decreased the LPP during 2,500–4,000 ms induced by the fear stimulus and the sadness stimulus (Wei et al., 2021). However, in our study, regulation of the down-regulation reappraisal and expressive suppression conditions (compared to the view condition) significantly decreased the LPP during 300–600 ms, which appeared to indicate the two strategies worked earlier in sadness regulation compared to the researches by Cheng et al. (2011) and Wei et al. (2021).

According to Gross (1998b), the various strategies of emotion regulation have different time courses in influencing emotion-generative processes. We found that the significant decreases at P2, P3a, and P3b (relative to the view condition) were induced by expressive suppression, while there were no significant differences in these components between the down-regulation reappraisal and the view condition. Reappraisal may require reinterpreting a situation’s meaning and time-consuming attention (Gross, 1998b), but expressive suppression may cost less attention resources in the early processing of sad images than under the down-regulation reappraisal and view conditions, which is probably linked to diverting attention resources away from the stimuli process for the masking and monitoring of emotional displays (Richards and Gross, 2000; Richards et al., 2003; Dillon et al., 2007). We also found that the enhancement at P2 and P3a (relative to expressive suppression) was induced through down-regulation reappraisal after image onset; both strategies significantly lowered the parietal LPP amplitude beginning at 300 ms. According to Gross’s theory of emotion regulation, cognitive reappraisal is an antecedent-focused strategy of emotion regulation, while expressive suppression involves suppressing emotional activities (such as facial expression) that will occur or are occurring and is a response-focused emotion regulation strategy (Gross, 1998a; Gross and Thompson, 2007; Chiao, 2011). Reappraisal required the participants to reinterpret emotion-inducing events, especially at the beginning of image presentation, when they needed to identify the images and change their meaning *via* the cognitive process. Therefore, reappraisal primarily involves more resources for cognitive reconstruction. However, expressive suppression required the participants to mask their facial expression and keep a neutral face when feeling the sadness of the protagonist in the images. Hence, expressive suppression mostly entails suppressing emotional activities (facial expression) but not sad emotion-induced processes. In this process, the participants felt sadness while masking their facial expression, which may have diverted some cognitive resources from cognitive processes to images (decreased P2, P3a, and P3b) and suppressed sad emotions; this objectively reduced sadness (attenuated parietal LPP) and impaired the recognition of sad images under the expressive suppression condition.

We performed further in-depth analysis and discovered that, contrary to our hypothesis, expressive suppression did not significantly reduce subjective feelings of sadness, although expressive suppression attenuated LPP amplitudes during 300–600 ms after image onset, which means that expressive suppression is not as effective as cognitive reappraisal in regulating sadness. Previous studies have also found that reappraisal more effectively reduced individuals’ negative subjective feelings for disgust than expressive suppression (Gross, 1998a; Goldin et al., 2008; Butler and Gross, 2009). According to Gross’s theory of emotion regulation (1998a), focusing on the response when the emotion is already fully activated, is more expensive than focusing on early regulation. In this study, expressive suppression primarily regulated the activated sadness response when the participants changed their behavioral expression only (masking their facial expression while keeping a neutral face), but not changed their perception to stimuli, so it only alleviated the intensity of sadness to a certain extent (for LPP but not for subjective feelings). As such, down-regulation reappraisal is a more effective strategy for sadness regulation than expressive suppression. A related explanation for this discrepancy between subjective ratings and physiological responses may also be tied to the intensity of sadness. Related studies argued that the experience of sadness was only modestly associated with physiological responses, suggesting that self-reported emotions do not inevitably correspond with simultaneously acquired physiological changes (Mauss et al., 2005). Another related ERP study about reappraisal and expressive suppression regarding negative emotions revealed a conflict between subjective ratings and the ERP outcomes. The self-reported results suggested effective down-regulation *via* reappraisal versus suppression, but the ERP data showed more reduction of LPP through suppression than reappraisal (Wang et al., 2018). The type and intensity of sadness elicited might contribute to the incongruity, and response coherence would rise as the intensity of emotion increases. Strong emotions may lead to greater coordination, but weak emotions might induce little coordination of response systems (Davidson, 1992; Mauss et al., 2005). Sadness had relatively moderate emotional intensity, which may have produced an incongruity between subjective ratings and the LPP results in our study.

Behavioral results on recognition demonstrate that expressive suppression significantly reduced the recognition accuracy of the sad images and slowed their response time as we expected, while down-regulation reappraisal only significantly slowed the response time of the sad images (not in line with our expectation). Thus, both down-regulation and expressive suppression impaired the participants’ recognition of sad images relative to the view condition, and expressive suppression had a more harmful effect on the recognition of sad images than down-regulation reappraisal. The impairment effect, *via* expressive suppression in the recognition of sad images, is consistent with the results of prior studies (Richards and Gross, 2000; Bonanno et al., 2004; Dillon et al., 2007; Hayes et al., 2010; Binder et al., 2012). Moreover, engaging in suppression to mitigate sad emotions may reduce memory for visual elements of emotional stimuli by biasing attentional resources away from elaborating on them in order to mask emotional displays (Richards and Gross, 2000; Richards et al., 2003; Dillon et al., 2007; Knight and Ponzio, 2013), confirmed by decreased P2, P3a, and P3b induced through expressive suppression versus view-sad condition. Contrary to our expectations, we also found that down-regulation reappraisal impaired the participants’ recognition of sad images, which was inconsistent with enhanced memories by cognitive reappraisal in some previous studies (Richards et al., 2003; Dillon et al., 2007; Hayes et al., 2010; Kim and Hamann, 2012; Yeh et al., 2019); however, some prior studies were showing impaired recognition for negative stimuli by down-regulation reappraisal (Knight and Ponzio, 2013; Leventon and Bauer, 2015), but in our study, the impairment by down-regulation reappraisal was less than that by expressive suppression because down-regulation did not reduce the participants’ Prs of sad images. Cognitive reappraisal may modulate memory through elaboration, attentional deployment, and arousal (Knight and Ponzio, 2013). Reduced sadness arousal to sad scenes (confirmed by attenuated LPP during 300–600 ms, 600–1,000 ms, and 1,000–1,500 ms) by the down-regulation reappraisal may cause an impairment of recognition of sad images. Whereas, reinterpreting a situation’s meaning may lead to a degree of attention and elaboration on sad stimuli, which was confirmed by the equal P2, P3a, and P3b between under the down-regulation and the new condition, as well as the increased P2 and P3a induced through down-regulation versus expressive suppression. However, the three together, a degree of attention, elaboration, and reduced arousal on sad stimuli, may lead to an impairment to a certain extent (only in the response time of recognition of sad images) by down-regulation reappraisal.

The limitations of this study should be considered when discussing the findings in relation to future research. For instance, facial muscle activity could be recorded to clarify whether participants suppress their emotional expression following the instructions, and to verify the regulation effects of cognitive reappraisal and expression suppression on sadness together with subjective experiences and ERP measures. Another example is that for this study, only immediate recognition of stimuli after emotion regulation was used, and the long-term effects of regulation strategies on memory should be surveyed after a long interval in future research. Additionally, we recruited only young undergraduate and graduate students, which do not represent the overall population, and other population should be surveyed to explore the effects of two emotion regulation strategies on sadness in future studies. Furthermore, gender differences in the effect of cognitive reappraisal and expression suppression on sadness and the recognition of sad scenes could be further explored in future studies.

## Conclusion

We explored the effects of cognitive reappraisal and expressive suppression on the regulation of sadness and the recognition of sad scenes. Down-regulation successfully reduced the LPP from 300 ms to 1,500 ms after image onset and self-reported sadness, while expressive suppression only successfully reduced the parietal LPP only during 300–600 ms but not in subjective ratings. Down-regulation reappraisal works lasting longer and is more effective than expressive suppression in sadness regulation. Both down-regulation and expressive suppression impaired the participants’ recognition of sad images, and expressive suppression had a more harmful effect in the recognition of sad images than down-regulation reappraisal. Our findings indicate that cognitive reappraisal and expressive suppression can effectively reduce sadness and impair memory in regulated sad scenes, and down-regulation reappraisal (relative to expressive suppression) is a more effective strategy in sadness regulation and has a somewhat less damaging effect in the recognition of sad images.

## Data availability statement

The original contributions presented in the study are included in the article/Supplementary material, further inquiries can be directed to the corresponding author/s.

## Ethics statement

The studies involving human participants were reviewed and approved by the Institutional Ethics Committee of Xinxiang Medical University in China. The patients/participants provided their written informed consent to participate in this study.

## Author contributions

CY supervised the project and designed the experiment. QD and CY collected the experimental data and prepared Figures 1–5 and Tables 1–3. CY and QD analyzed experimental data and wrote the main text. CY, YW, MW, TG, and XL reviewed the manuscript. All authors contributed to the article and approved the submitted version.
