# Supplementary material for: The effect of cognitive reappraisal and expression suppression on sadness and the recognition of sad scenes: An event-related potential study
Source: Front Psychol. 2022 Sep 23;13:935007. doi: 10.3389/fpsyg.2022.935007 (PMC9537681; doi:10.3389/fpsyg.2022.935007)
Supplement: Supplementary file 1 [file Data_Sheet_1.docx]

# Materials

Two-way, 3 (down-regulation, view-sad, and expressive suppression) × 2 (old/new: new images and old images) repeated-measures ANOVAs were conducted on the valence, arousal, and sadness ratings of sad images. The ANOVAs revealed that the main effects of the regulation strategy were not significant [valence: *F* _(2, 174)_ < 0.01, *p* = 0.997, *η_p_*^2^ < 0.001; arousal: *F* _(2, 174)_ < 0.001, *p* = 1.000, *η_p_*^2^ < 0.001; sadness: *F* _(2, 174)_ < 0.01, *p* = 0.996, *η_p_*^2^ < 0.001], and the main effects of old/new type were not significant [valence: *F* _(1, 174)_ = 0.02, *p* = 0.903, *η_p_*^2^ < 0.001; arousal: *F* _(1, 174)_ = 0.04, *p* = 0.846, *η_p_*^2^ < 0.001; sadness: *F* _(1, 174)_ = 0.04, *p* = 0.849, *η_p_*^2^ < 0.001], and there were no significant interactions between the regulation strategy and old/new type [valence: *F* _(2, 174)_ = 0.01, *p* = 0.993, *η_p_*^2^ < 0.001; arousal: *F* _(2, 174)_ < 0.001, *p* = 1.000, *η_p_*^2^ < 0.001; sadness: *F* _(2, 174)_ < 0.01, *p* = 0.999, *η_p_*^2^ < 0.001], suggesting that these six (3*2) groups of sad images had equal valence, arousal, and sadness ratings (see **Table 1**). The neutral images were used in the view condition, and there were no significant differences in the valence and arousal ratings between the old and new groups of neutral images [valence: *t* _(58)_ = -0.07, *p* = 0.948; arousal: *t* _(58)_ = 0.01, *p* = 0.991].
